# 2260: Silicone renal tumor models: The validation of a surgical training tool

**DOI:** 10.1017/cts.2017.168

**Published:** 2018-05-10

**Authors:** Steven Monda, Jonathan R. Weese, Barrett G. Anderson, Ramakrishna Venkatesh, Baisong Cheng, Robert S. Figenshau

## Abstract

OBJECTIVES/SPECIFIC AIMS: More partial nephrectomies are performed every year as a surgical treatment for kidney cancer. However, this procedure remains technically challenging. Surgeons require a substantial number of cases before their performance plateaus. No established practice mode exists; thus, there is a need for training models to simulate real tumor excisions and kidney suturing. In this study, we seek to validate these silicone models using multiple simulations with urologists of different training levels. METHODS/STUDY POPULATION: We created silicone renal tumor models using 3D printed molds of a patient’s kidney with a mass. Medical students, urology residents, fellows, and attending surgeons are recruited to perform simulated partial nephrectomies on these models. Four trials are performed with a da Vinci surgical robot on 2 different days. We are evaluating surgeon performance and improvement using validated measures as well as operation-specific metrics. Operation-specific metrics include renal artery clamp time and surgical margins. Validated measures of self-assessed operative demand (NASA TLX) and reviewer-assessed surgical performance (GEARS) are also recorded across trials. RESULTS/ANTICIPATED RESULTS: The preliminary results of 2 medical students, 10 urology residents, 3 endourology fellows, and 2 attending urologists are reported here. Model face validity was evaluated on a 0–100 sliding scale anchored at unrealistic and realistic. Mean results thus far are 77.7 for overall feel, 82.7 for needle driving, 75.6 for cutting, and 73.2 for visual representation. Between trials 1 and 4 there was a mean reduction of 3.26 minutes in renal artery clamp time, and a 75% reduction in positive margins. There was a reduced incidence of positive surgical margins with advanced training stage. Fellows, residents, and medical students had positive tumor margins in 25%, 50%, and 75% of their trials, respectively. We expect to recruit 15 additional subjects for this study. Upon completion of data acquisition, more robust statistical comparisons and measures will be reported. DISCUSSION/SIGNIFICANCE OF IMPACT: Face validity measures indicate the model adequately represents reality. Preliminary data suggest improved surgical performance over the course of the training and better performance in urologists of higher training levels. This model may have potential for broader application and integration into minimally invasive surgery training programs.

